# Drug metabolite synthesis by immobilized human FMO3 and whole cell catalysts

**DOI:** 10.1186/s12934-019-1189-7

**Published:** 2019-08-12

**Authors:** Chongliang Gao, Tingjie Zheng

**Affiliations:** Department of Life Sciences and Systems Biology, University of Torino, Via Accademia Albertina 13, 10123 Turin, Italy

**Keywords:** Flavin-containing monooxygenase isoform 3, Glucose dehydrogenase, Enzyme immobilization, Whole cell catalysis, Benzydamine

## Abstract

**Background:**

Sufficient reference standards of drug metabolites are required in the drug discovery and development process. However, such drug standards are often expensive or not commercially available. Chemical synthesis of drug metabolite is often difficulty due to the highly regio- and stereo-chemically demanding. The present work aims to construct stable and efficient biocatalysts for the generation of drug metabolites in vitro.

**Result:**

In this work, using benzydamine as a model drug, two easy-to-perform approaches (whole cell catalysis and enzyme immobilization) were investigated for the synthesis of FMO3-generated drug metabolites. The whole cell catalysis was carried out by using cell suspensions of *E. coli* JM109 harboring FMO3 and *E. coli* BL21 harboring GDH (glucose dehydrogenase), giving 1.2 g/L benzydamine *N*-oxide within 9 h under the optimized conditions. While for another approach, two HisTrap HP columns respectively carrying His_6_-GDH and His_6_-FMO3 were connected in series used for the biocatalysis. In this case, 0.47 g/L benzydamine *N*-oxide was generated within 2.5 h under the optimized conditions. In addition, FMO3 immobilization at the C-terminal (membrane anchor region) significantly improved its enzymatic thermostability by more than 10 times. Moreover, the high efficiency of these two biocatalytic approaches was also confirmed by the *N*-oxidation of tamoxifen.

**Conclusions:**

The results presented in this work provides new possibilities for the drug-metabolizing enzymes-mediated biocatalysis.

**Electronic supplementary material:**

The online version of this article (10.1186/s12934-019-1189-7) contains supplementary material, which is available to authorized users.

## Background

Human FMO3 is an important non-cytochrome P450 drug metabolizing enzyme in adult human liver, catalyzing the monooxygenation of a wide variety of nucleophilic heteroatom-containing drugs, xenobiotics and dietary compounds to their corresponding *N*-oxide metabolites [1]. In the course of drug discovery and development process, sufficient reference standards of drug metabolites are required for their pharmacologic and toxicologic characterization [2]. However, such drug standards are often expensive or not commercially available, particularly in the case of preclinical/clinical or new therapeutic drugs. Chemical synthesis of drug metabolites is often difficulty due to the highly regio- and stereo-chemically demanding [3]. In addition, although drug metabolites can be generated directly by drug metabolizing enzymes in vitro with high selectivity, the synthesis on preparative scale is challenging due to low catalytic activity/protein stability, difficult re-use of the enzyme, and requirement for the expensive cofactor(s). These drawbacks can often be overcome by whole cell catalysis due to advantages like low cost, large scale application and cofactor regeneration [4–6], as well as enzyme immobilization due to advantages such as improved enzyme thermostability, feasible enzyme recovery/recycling, and straightforward downstream processing [7, 8].

**Scheme 1:**
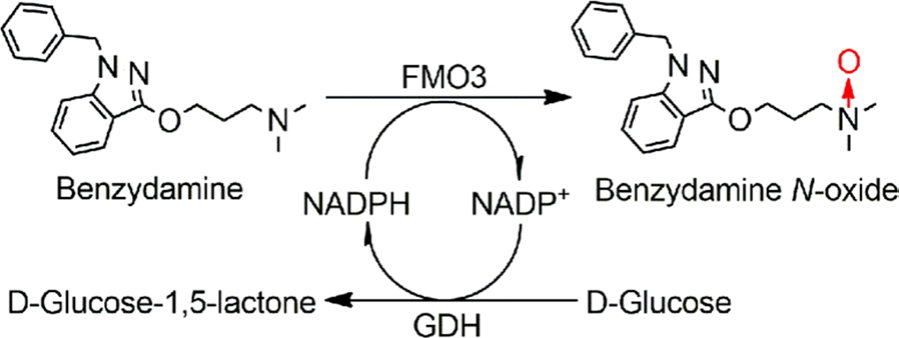
FMO3-mediated catalysis converting benzydamine to its *N*-oxide metabolite. GDH-mediated glucose oxidation was used for NADPH regeneration

Benzydamine is a nonsteroidal anti-inflammatory drug [9], and primarily converted into *N*-oxide metabolite by FMO3 in human liver. The *N*-oxygenation of benzydamine as a marker reaction can reflect FMO3 activity [10, 11]. In this work, using benzydamine as a model drug, two approaches were tested and optimized for the synthesis of benzydamine *N*-oxide: an enzyme immobilization system using two HisTrap HP columns connected in series carrying His_6_-GDH and His_6_-FMO3 enzymes respectively, and a two-strain-mixed-culture strategy using cell suspensions of *E. coli* JM109 (containing a FMO3 gene) and BL21 (containing a GDH gene). GDH-mediated glucose oxidation is used for recycling the cofactor NADPH (Scheme 1).

## Results and discussion

### Purification and spectroscopic characterization of FMO3 and GDH

FMO3 and GDH were successfully expressed in *E. coli* and purified by Ni-affinity chromatography. Subsequently, spectroscopic characteristics of the purified proteins were determined. UV–vis absorption spectra show three maxima around 280, 375 and 450 nm for the purified FMO3, and one maximum around 280 nm for the purified GDH (Additional file 1: Fig. S1a, b). The concentration of holo-FMO3 (168.3 μM, determined by absorbance at 450 nm) accounts for 56% of the total FMO3 (both holo- and apoprotein, 300.3 μM, determined by Bradford assay). In addition, the SDS-PAGE (Additional file 1: Fig. S1c) shows a band around 58 kDa for FMO3 and 30 kDa for GDH in good agreement with their molecular weights calculated from the amino acid sequences.

### Catalytic properties of FMO3 and GDH

**Fig. 1:**
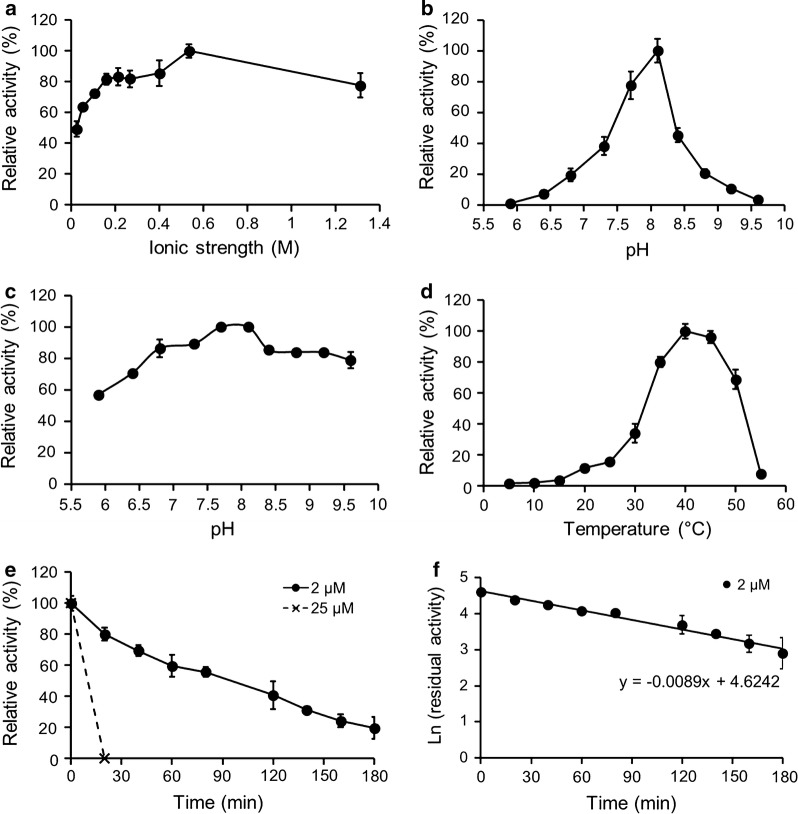
Effects of **a** ion strength on FMO3-mediated benzydamine *N*-oxygenation. Effects of pH on FMO3 **b** activity and **c** stability. **d** The dependence of FMO3 activity on temperature. **e** Thermostability of FMO3 at two different pre-incubation concentration (2 and 25 μM), and the data were fitted to **f** the first order plot. The maximum activity was taken as 100%

**Fig. 2:**
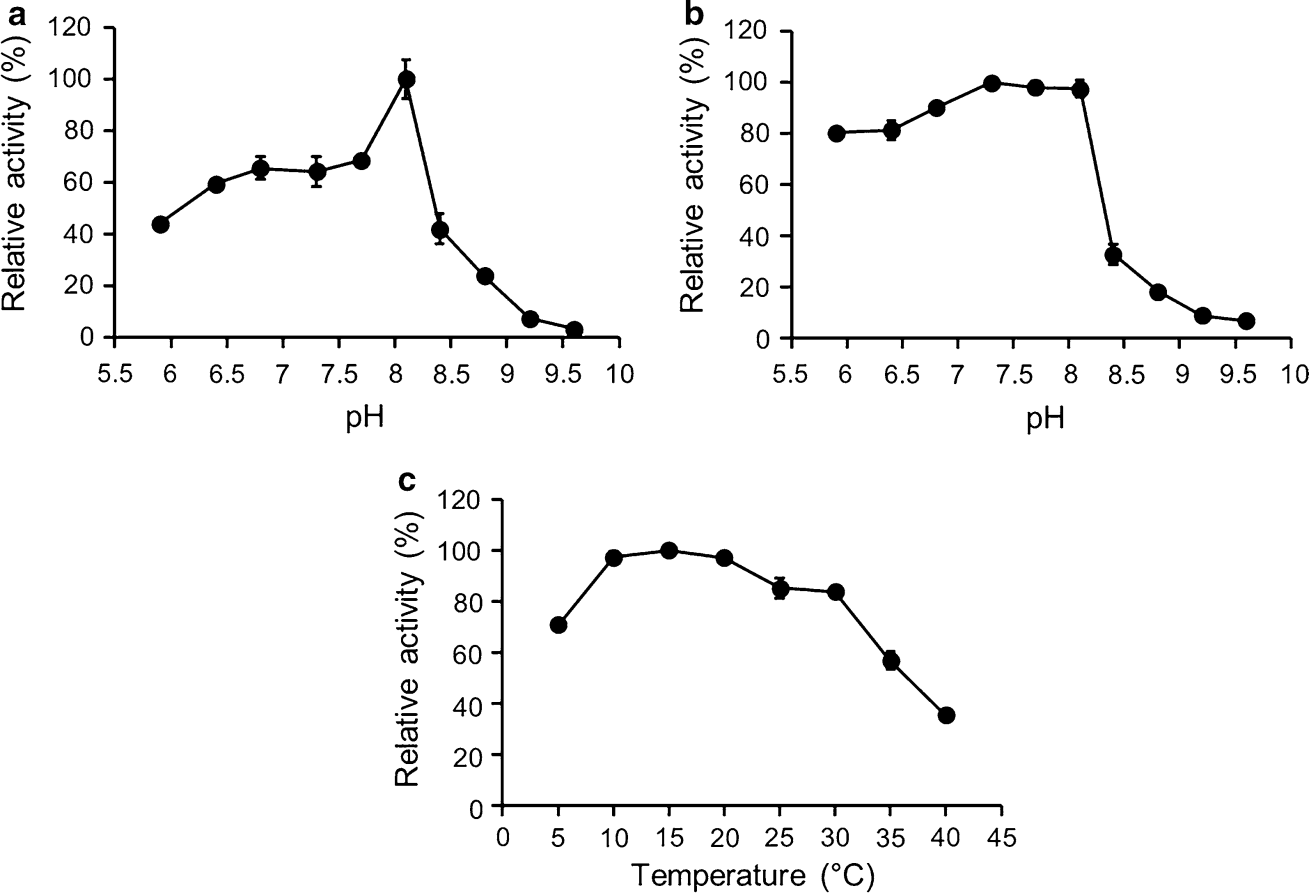
Effects of pH on GDH **a** activity and **b** stability. **c** Dependence of GDH activity on temperature. The maximum activity was taken as 100%

Subsequently, the catalytic properties of the purified enzymes were characterized in term of ion strength, pH, pH stability, temperature and thermostability. Figure 1a, b shows that the highest activity of FMO3 is achieved at pH 8.1 with an optimized ionic strength of 0.535 M, and this enzyme is stable in alkaline environments (Fig. 1c). In addition, the optimum temperature of FMO3 activity is 40 °C (Fig. 1d), and a relatively weak activity is observed at temperatures below 25 °C. The half-life of FMO3 at 40 °C is determined based on the formula of *T*_1/2_ = ln2/*k*_d_, where *k*_d_ is calculated from the slope of the plot ln (residual activity) versus time [12]. Figure 1e, f and Table 1 show that FMO3 has a half-life of 78 min at a low preincubation concentration (2 μM). However, this enzyme is unstable and totally inactivated within 20 min at a high preincubation concentration (25 μM). For the characterization of GDH-mediated glucose oxidation, Fig. 2 shows an optimal pH of 8.10 and temperature of 15 °C. Unlike FMO3, GDH is stable in acidic environments and has a relatively high activity at low temperatures.

**Table 1:** Thermostability of FMO3 at different enzyme concentration at 40 °C

Enzyme	Enzyme concentration (μM)	*t*_1/2_, min
Free FMO3	2	78
Free FMO3	25	< 20
Immobilized FMO3	25^a^	289

^a^μM/L of column volume

### Immobilization of enzymes

Following enzymatic characterizations of the purified FMO3 and GDH, enzyme immobilizations were carried out. The His_6_-tagged FMO3 (with a total activity of 693 mU) and GDH were separately loaded onto two different HisTrap HP columns (1 mL) at a flow rate of 0.5 mL/min by using a peristaltic pump. Immobilization was completed as soon as the enzyme solution was passed through the affinity column (enzyme purification and immobilization can be combined here). The immobilization yield was determined by measuring the total residual enzyme activity in the flow-through [13]. Table 2 shows that more than 99% of immobilization yield is achieved for both GDH and FMO3.

**Table 2:** Specific activity, immobilization yield and activity recovery of GDH and FMO3

	Specific activity^a^ (U/g)		Immobilization yield (%)	Activity recovery (%)
	Before immobilization	After immobilization		
GDH	712	332	> 99	46
FMO3	462	173	> 99	37

^a^One unit (U) of enzyme activity is defined as the amount of enzyme required to release 1 μM of substrate per minute. The specific activity was determined at 35 °C in 50 mM Tris–HCl, pH 8.4. The immobilization yield and activity recovery were determined according to [13]

### Optimization of the immobilized-enzyme system

**Fig. 3:**
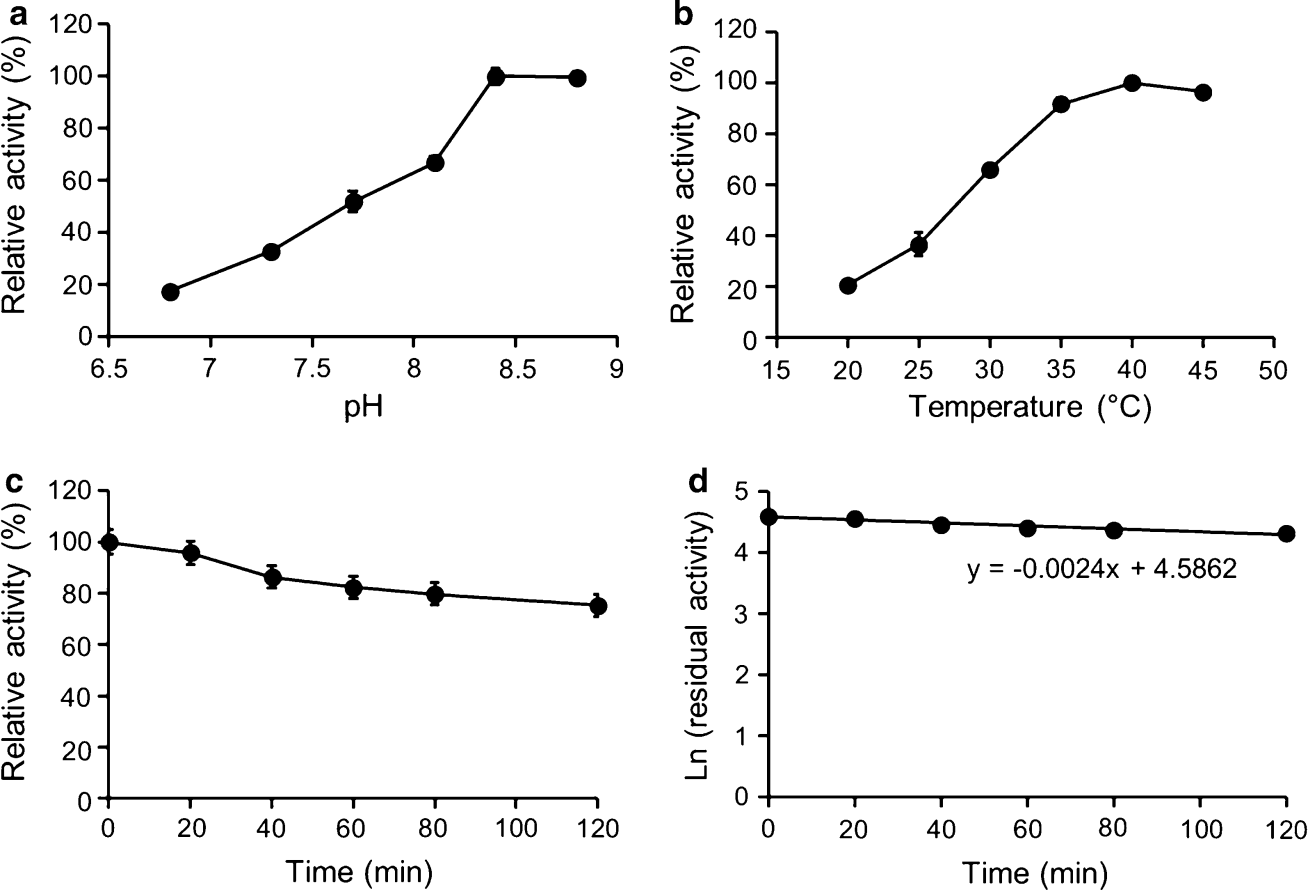
Determination of the optimal **a** pH and **b** temperature of the immobilized GDH-FMO3 enzyme system. **c** Thermostability of the immobilized FMO3 (25 μM/L of column volume), and the data were fitted to **d** the first order plot. The maximum activity was taken as 100%

**Fig. 4:**
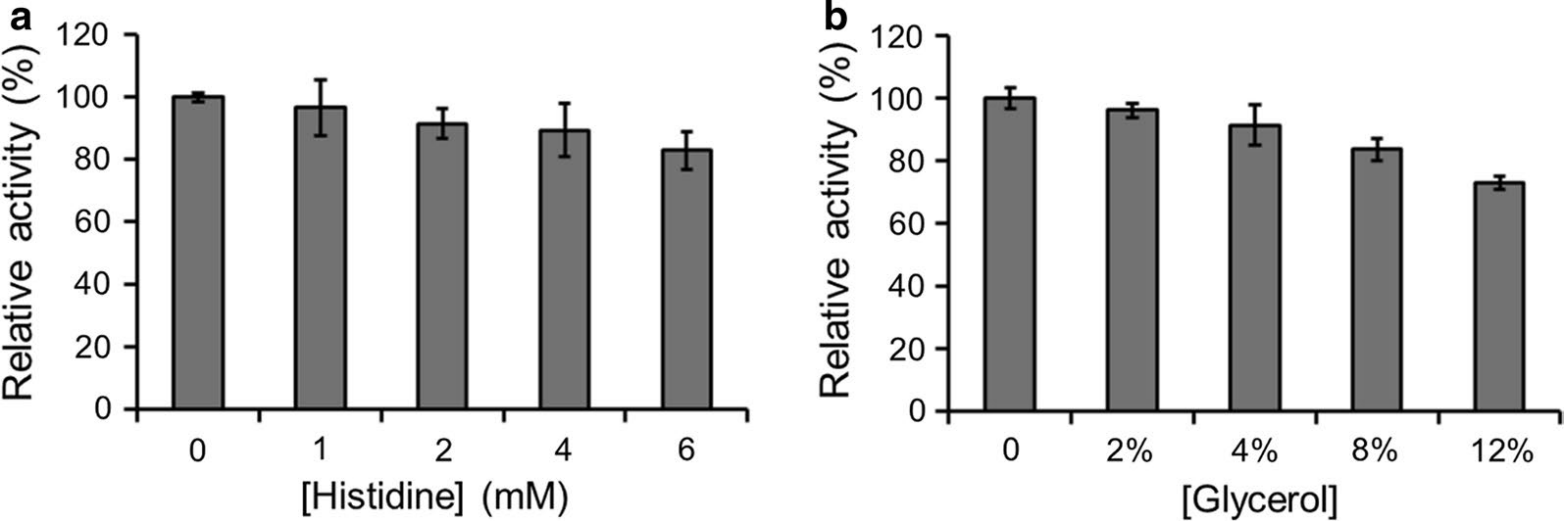
Effects of different concentrations of **a** histidine and **b** glycerol in substrate solution on the conversion of benzydamine to its *N*-oxide metabolites using the immobilized GDH-FMO3 system. The maximum activity was taken as 100%

Subsequently, the two enzyme columns were connected in series used for the biotransformation (Additional file 1: Fig. S2). The experiments were performed in a Stuart incubator, and the effects of pH, temperature, histidine and glycerol addition on the substrate conversion were investigated (Figs. 3 and 4). Figure 3a, b shows that the highest activity was obtained at pH 8.4 and 40 °C. In addition, FMO3 immobilization significantly increased its thermostability by more than 10 times (Fig. 3c, d and Table 1). If the inactivation of FMO3 was induced by intermolecular hydrophobic interaction between the C-terminal (membrane anchor region) [14], the improved thermostability of FMO3 after affinity immobilization, would be readily rationalized.

Another parameter to be optimized was the histidine concentration in substrate mixture. Although a certain concentration of histidine in solution can decrease non-specific interactions of enzymes to the affinity column [15, 16], the activity of immobilized FMO3 was not improved after the addition of histidine (Fig. 4a). Similar results were also observed for the addition of glycerol in the substrate mixture (Fig. 4b).

### Synthesis of benzydamine *N*-oxide by the immobilized-enzyme system

**Fig. 5:**
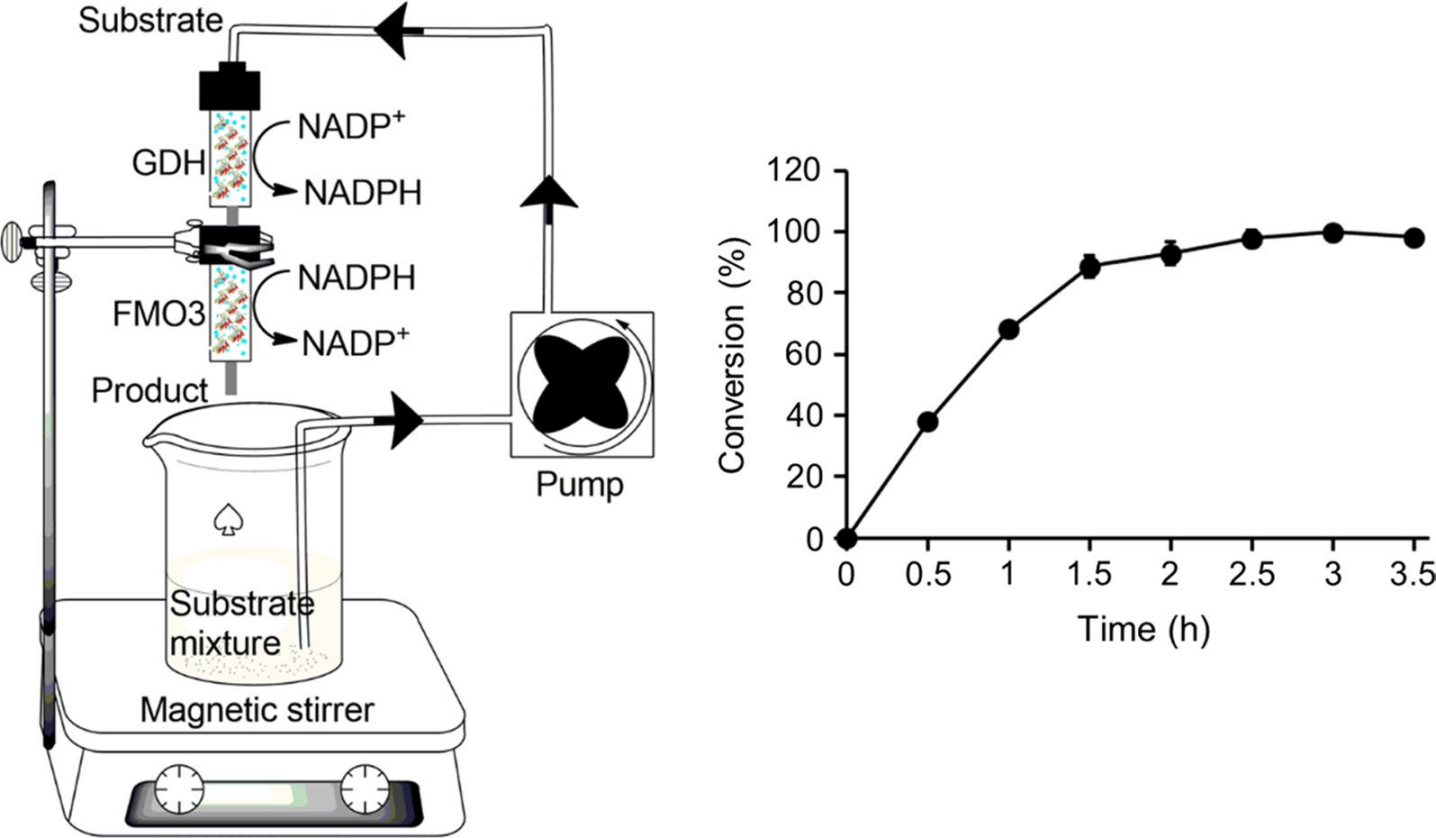
Under the optimized conditions, biotransformation of benzydamine to its *N*-oxide metabolite by the enzyme immobilization approach. The substrate mixture was circularly flowed through the enzyme columns

Following the optimization of the different parameters, the GDH-FMO3 columns were used for the biotransformation of benzydamine to its *N*-oxide metabolite. Table 2 shows an activity recovery of 46% and 37% for the enzyme immobilization of GDH and FMO3 respectively. Figure 5 shows that more than 97% conversion (above 0.47 g/L *N*-oxide product) was reached within 2.5 h under the optimized conditions.

### Synthesis of benzydamine *N*-oxide by the optimized whole cell system

**Fig. 6:**
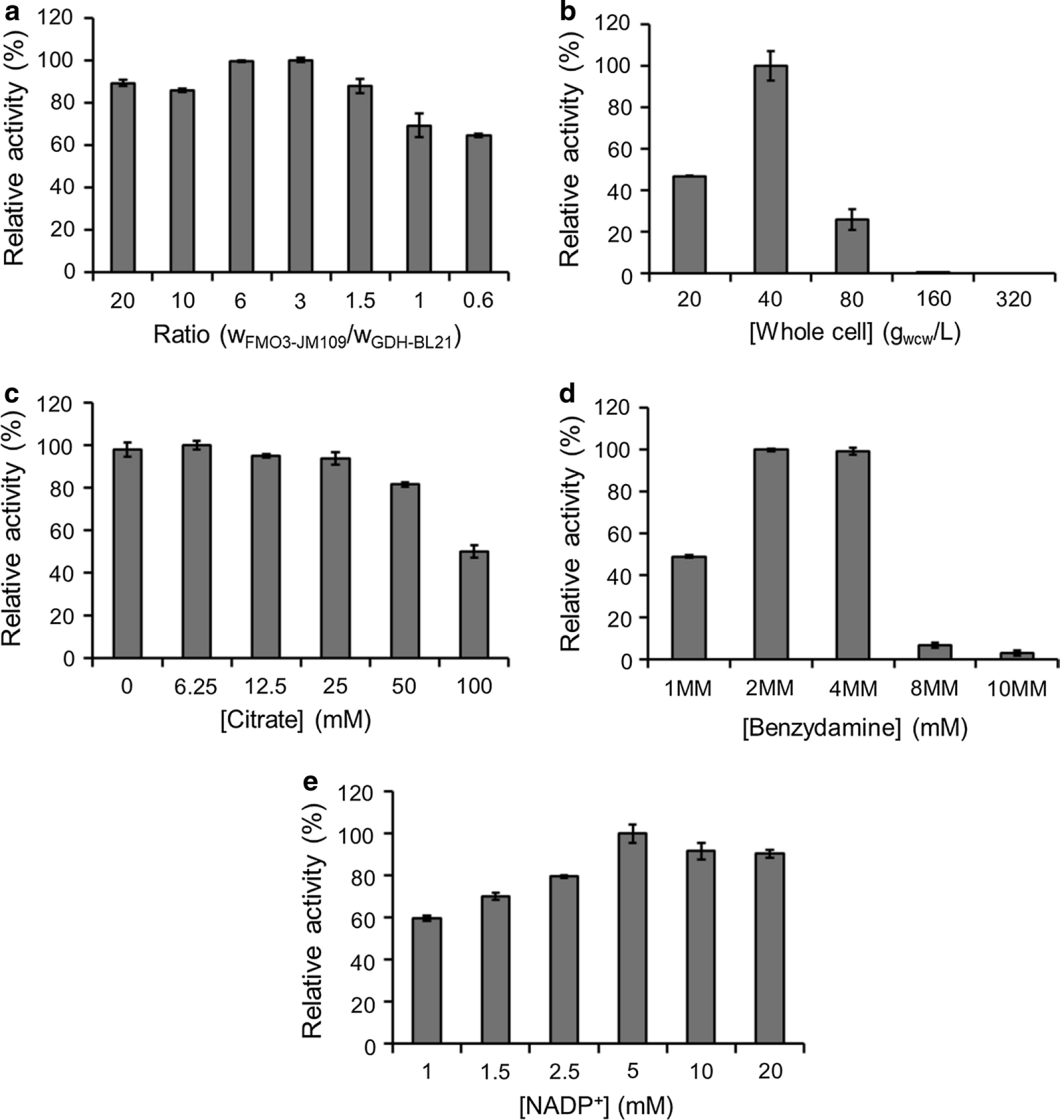
**a** Determination of the optimal ratio (wet cell weight) of FMO3-containing *E. coli* JM109 and GDH-containing *E. coli* BL21. Optimizing the **b** cell concentration (with a constant W_FMO3-JM109_/W_GDH-BL21_ ratio of 3) and investigating the effects of **c** citrate addition, **d** benzydamine concentration and **e** NADP^+^ concentration on biotransformation. The maximum activity was taken as 100%

**Fig. 7:**
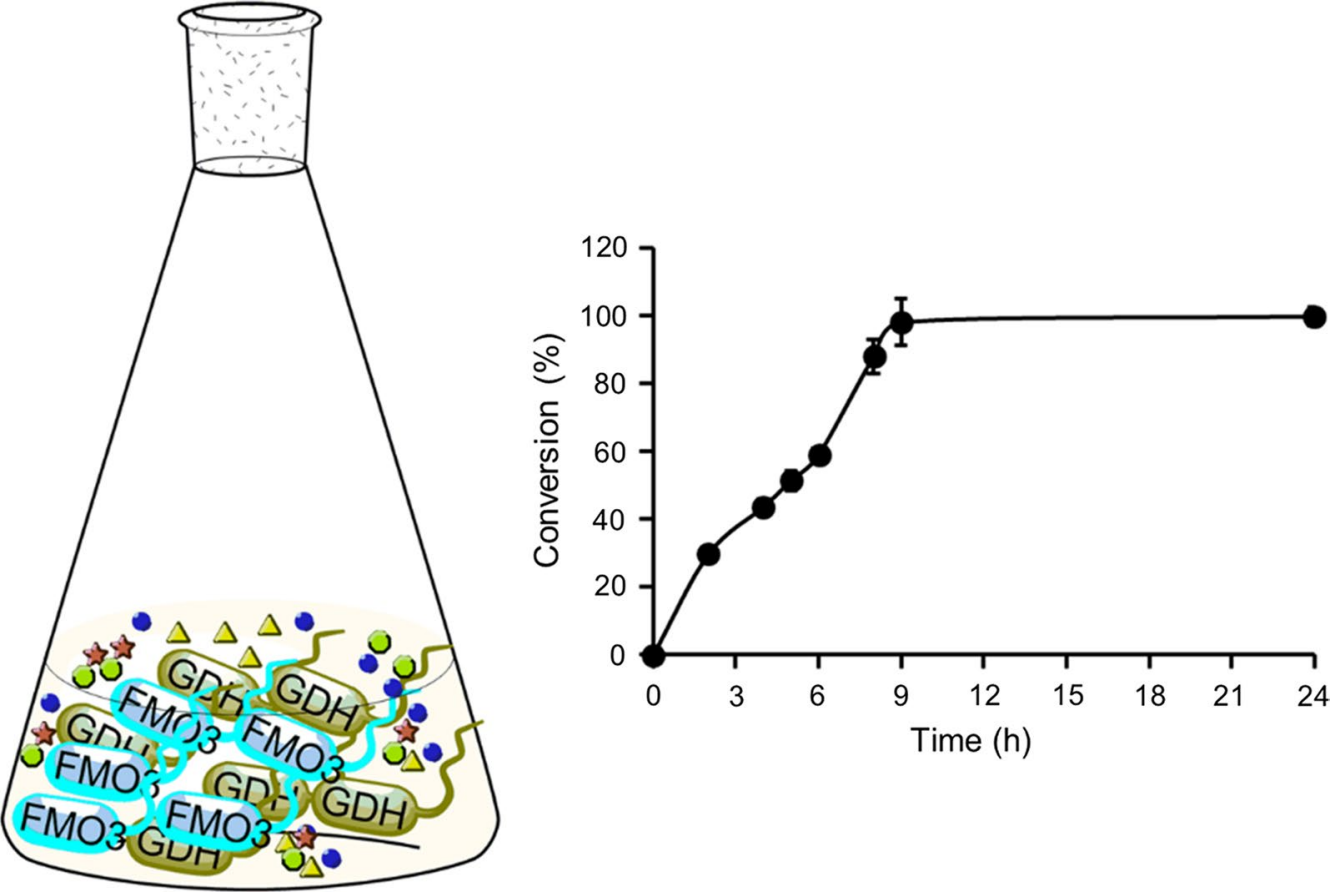
Under the optimized conditions, biotransformation of benzydamine to its *N*-oxide metabolite was performed by using the two-strain-mixed-culture approach

Another approach is whole cell catalysis. A two-strain-mixed-culture system was tested for the biotransformation of benzydamine to its *N*-oxide metabolite. Effects of the ratio of two cell suspensions (harboring either FMO3 or GDH), total cell density, citrate, substrate and NADP^+^ concentration on biotransformation was investigated (Fig. 6). Figure 6a, b show that the highest conversion is achieved at a FMO3/GDH ratio of 3 with a total cell concentration of 40 g_wcw_/L. Although citrate was previously proved to accelerate the NADPH regeneration within *E. coli* [17], its addition did not improve the biotransformation here (Fig. 6c). Moreover, substrate inhibition was observed at a high concentration (above 8 mM) of benzydamine (Fig. 6d), and the addition of 5 mM NADP^+^ gave the highest FMO3 activity (Fig. 6e). Under the optimized conditions, more than 98% conversion (above 1.2 g/L *N*-oxide product) was reached within 9 h (Fig. 7). Whole cell catalysis was also performed in the absence of GDH but presence of FMO3, and only limited benzydamine *N*-oxide (< 5% conversion yield) was generated within 24 h. Cells harboring the empty vector (in the absence of FMO3) served as negative control and as expected negligible benzydamine conversion (< 1%) was observed within 24 h.

In the specific case of FMO enzymes, Hanlon and co-workers have successfully applied the FMO3-based whole cell catalysis for the conversion of Moclobemide to its *N*-oxide metabolite with a product titer of 0.086 g/L within 24 h [17]. In addition, in another case of FMO2-based whole cell catalysis, 100% conversion (around 0.325 g/L product titer) of 1 mM benzydamine was reached within 16 h [18]. In both cases above, the addition of NADP^+^ and citrate was used for the NADPH regeneration. For the enzyme immobilization, Ramana and co-workers have successfully improved the stability of FMO3 by immobilizing this enzyme on magnetic nanoparticles using glutaraldehyde as cross-linker. The immobilization of this enzyme onto the glutaraldehyde-coated nanoparticles was instantaneous, with an immobilization yield of 100% [19]. FMO3 has also been successfully immobilized on electrode surfaces, in order to be used for electrocatalysis or developing electrochemical biosensors [20].

The approaches described herein provide new possibilities for the FMO3-mediated biotransformation. Affinity immobilization of the membrane anchor region of FMO3 leads to significantly increased thermostability, providing a strategy for biotechnological applications of membrane-bound proteins and drug-metabolizing enzymes.

### Synthesis of tamoxifen *N*-oxide by the two easy-to-perform approaches

**Fig. 8:**
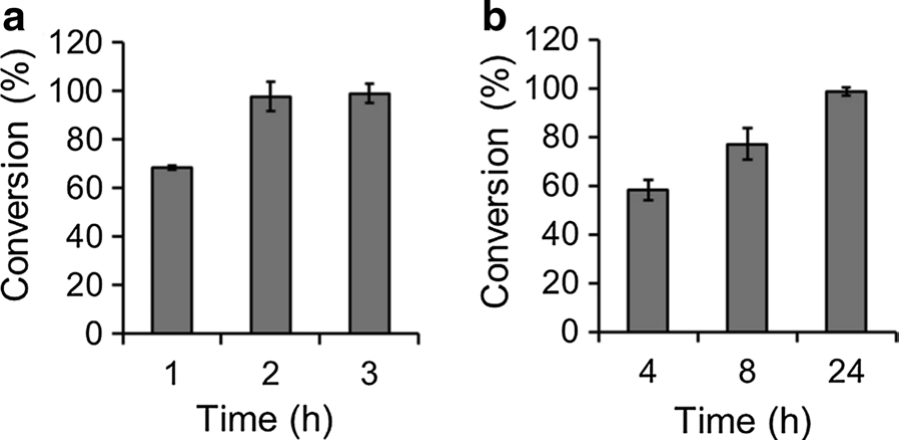
Biotransformation of tamoxifen to its *N*-oxide metabolite by the **a** immobilized enzymes and **b** whole cell catalysts

In order to further confirm the efficiency of the two biocatalytic systems. Another FMO3 substrate, tamoxifen, was tested. The results showed that more than 95% of substrate conversion (0.19 g/L *N*-oxide product) was obtained within 2 h using the immobilized enzyme system (Fig. 8a). The whole cell catalysis showed 77% (0.30 g/L *N*-oxide product) and 100% (0.38 g/L *N*-oxide product) of substrate conversion within 8 and 24 h respectively (Fig. 8b).

Although high conversion yields were obtained for the *N*-oxidation of both benzydamine and tamoxifen in this work, substrate solubility and substrate/product inhibition should be tackled in the future. For FMO3, majority of its known substrates are hydrophobic compounds with low solubility or even insolubility in aqueous media, which greatly limits the enzymatic efficiency. One solution to this limitation is the use of water miscible or immiscible organic solvents to develop mono- or biphasic media for efficient biocatalysis. Water miscible solvents are generally used to facilitate the substrate/product solubility and to improve the reaction rates whereas water immiscible solvents can regulate the distribution of toxic substrates/products around the enzyme, relieving substrate or product inhibition [21, 22]. In addition, overexpression of FMO3 in *E. coli* is another challenge which need to be tackled in order to further improve the catalytic efficiency of whole cell catalysts. Human FMO3 is a membrane associated protein. Overexpression of this protein is limited by the membrane’s capacity. C-terminal truncation of FMO3 can be a possible strategy to remove membrane-bound anchor and produce soluble cytosolic proteins.

## Conclusions

In summary, two easy-to-perform approaches were successfully applied for synthesizing FMO3-generated drug metabolites, and both methods lead to high substrate conversions. Future work will focus on relieving substrate inhibition during the whole cell catalysis.

## Methods

### Materials and chemicals

2-Mercaptoethanol, Acetonitrile, Ampicillin sodium salt, Benzydamine hydrochloride, Benzydamine *N*-oxide hydrogen maleate, Bradford Reagent, d(+)-Glucose, Flavin adenine dinucleotide disodium salt hydrate (FAD), HisTrap HP columns, IGEPAL CA-630, Imidazole, Kanamycin, Nickel(II) sulfate hexahydrate, Lysozyme from hen egg white, Phenylmethylsulfonyl fluoride, and potassium phosphate were purchased from Sigma-Aldrich. NADPH tetra(cyclohexammoninum) and β-NADP sodium salt were purchased from Carbosynth. Isopropyl-beta-d-thiogalactopyranoside (IPTG) was purchased from BIOSYNTH. TRIS Base was purchased from Fisher Molecular Biology. Yeast Extract and Tryptone were purchased from Fisher Bioreagents. PageRuler Unstained Protein Ladder was purchased from Thermo Fisher Scientific Baltics UAB.

### Protein expression

Human FMO3 gene with C-terminal His6-tags inserted into pJL2 was expressed in *E. coli* JM109 [23], and glucose dehydrogenase (GDH) constructed in pET28a was expressed in *E. coli* BL21 [24]. After 24 h post-induction, the cells were harvested by centrifugation at 4500×*g*, washed twice with 0.1 M phosphate buffer (pH 7.0), and stored at − 20 °C.

### Protein purification

For the purification of FMO3, cell pellets were resuspended in lysis buffer (20% glycerol, 5 mM β-mercaptoethanol, 0.5 mM PMSF, 0.5 mg/mL lysozyme in 50 mM potassium phosphate buffer, pH 7.4), and stirred at 4 °C for 1 h, followed by ultrasonication. The lysate was ultra-centrifuged at 41,000 rpm for 1 h at 4 °C. The resulting cell debris was resuspended in 1% IGEPAL CA-630, and stirred for 2 h at 4 °C, followed by ultra-centrifuged at 41,000 rpm for 1 h at 4 °C. The resulting supernatant was loaded onto a DEAE ion exchange column followed by Ni-affinity chromatography, and the target proteins were eluted by application of 40 mM Histidine. Eluted fractions detected by spectrophotometer were collected and buffer exchanged to storage buffer (100 mM phosphate buffer at pH 7.4, 20% glycerol and 1 mM EDTA) by 30 kDa cutoff Amicon membranes. The purified protein was analyzed by 12.5% SDS-PAGE and stored at − 80 °C. The holo-protein concentration was determined spectrophotometrically using the absorbance at 450 nm (with a molar extinction coefficient of 11,300 M^−1^ cm^−1^) [25]. The concentration of total protein (both holo- and apo-protein) was determined by Bradford assay.

For the purification of GDH, cell pellets resuspended in 0.1 M phosphate buffer (pH 7.4) were disrupted by sonication, followed by centrifugation at 12,000×*g* for 20 min at 4 °C. The resulting supernatant was loaded onto a nickel affinity column. Subsequently, the column was washed by washing buffer 1 (50 mM imidazole, 50 mM sodium phosphate buffer, pH 7.0, 300 mM NaCl), and washing buffer 2 (the concentration of imidazole was increased to 100 mM). The 6× His tagged proteins were eluted with 250 mM imidazole. The eluted solution containing target proteins was buffer exchanged to the storage buffer (0.1 M phosphate buffer, pH 7.0, 50% glycerol) by 30 kDa cutoff Amicon membranes, analyzed by 12.5% SDS-PAGE and stored at − 80 °C. The protein concentration was determined by Bradford assay.

### The effect of ionic strength on FMO3 activity

The optimal condition for FMO3-mediated catalysis was investigated, as well as GDH-mediated glucose oxidation.

First of all, the effects of ionic strength on FMO3-mediated catalysis was investigated. The reaction mixture contained 0.3 μM FMO3, 0.5 mM benzydamine (the substrate), 0.5 mM NADPH, in 0.01–0.5 M of potassium phosphate buffer (pH 7.4) with a total volume of 200 μL. The corresponding ion strength of the reaction solution was determined according to https://www.liverpool.ac.uk/pfg/Research/Tools/BuffferCalc/Buffer.html as shown in Table 3. The reaction was initiated by addition of NADPH and incubated at 35 °C for 15 min before termination by the addition of 200 μL ice-cold acetonitrile. The resulting mixture was centrifuged at 12,000×*g* for 5 min, and 100 μL of the supernatant was analyzed by HPLC equipped with 4.6 × 150 mm 5 μm Eclipse XDB-C18 column at room temperature with the UV–visible detector set at 308 nm. For the separation of benzydamine and its *N*-oxide, a mobile phase of 22% acetonitrile and 78% formic acid (0.1%) in water was used at a flow rate of 0.5 mL/min.

**Table 3:** preparation of phosphate buffer (pH 7.4) with different ionic strength

Concentration (M)	Ionic strength (M)
0.01	0.026
0.02	0.052
0.04	0.107
0.06	0.16
0.08	0.214
0.1	0.267
0.15	0.401
0.2	0.535
0.5	1.313

### pH dependence of FMO3

The optimal pH of FMO3-mediated benzydamine *N*-oxidation was investigated at different pH with a constant ion strength of 535 mM. The buffers were prepared according to https://www.liverpool.ac.uk/pfg/Research/Tools/BuffferCalc/Buffer.html. The reaction mixture contained 0.6 μM FMO3, 0.5 mM benzydamine, 1 mM NADPH, in buffers of different pH. The reaction mixture was incubated at 35 °C for 15 min before terminated by 200 μL ice-cold acetonitrile. The sample was analyzed by HPLC as described above.

### pH stability of FMO3

The pH stability of FMO3 was performed by incubating the enzyme (with a concentration of 6 μM) in different pH buffer for 12 h at 4 °C. The residual activity of FMO3 was determined at 35 °C, with a reaction mixture of 0.6 μM FMO3, 1 mM NADPH, 0.5 mM benzydamine, 0.2 M potassium phosphate buffer (pH 8.1), in a final volume of 200 μM. The reaction was stopped by 200 μL ice-cold acetonitrile and analyzed by HPLC as described above.

### Optimal temperature and thermostability of FMO3

The optimal temperature of FMO3-mediated catalysis was determined by measuring the activity at different temperatures ranging from 5 to 50 °C. The thermostability of FMO3 was determined by incubating the enzyme (with a concentration of 2 μM and 25 μM) at 40 °C for 3 h. Samples were taken at different time-points, followed by measuring residual activity at 35 °C as described above.

### Optimal pH and pH stability of GDH

Optimal pH of GDH-mediated catalysis was determined by using different pH buffer as described above (with a constant ion strength of 535 mM). The reaction mixture consisted of 0.2 μM GDH, 0.2 mM NADP^+^, 1 mM glucose, in different pH buffer with a total volume of 200 μL. The generated NADPH was monitored spectrophotometrically using the absorbance at 340 nm (with a molar extinction coefficient of 6, 220 M^−1^ cm^−1^). The pH stability of GDH was determined by pre-incubating the enzyme (with a final concentration of 2 μM) in different pH buffer for 24 h at 4 °C, followed by measuring residual activity at 25 °C in buffer of pH 8.1.

### Optimal temperature of GDH

The optimal temperature of GDH-mediated catalysis was determined by measuring the activity at different temperatures ranging from 5 to 40 °C. The reaction mixture consisted of 0.2 μM GDH, 0.2 mM NADP^+^, 1 mM glucose, in 0.2 M phosphate buffer (pH 8.1) with a total volume of 200 μL. The activity assay of GDH was performed by using spectrophotometer as described above.

### Enzyme immobilization—preparation of enzyme binding columns

The HisTrap™ HP column (1 mL, precharged with Ni^2+^ ions) washed with 100 mL of 0.2 M phosphate buffer (pH 8.1) was used for the enzyme immobilization. The His_6_ tagged GDH and FMO3 were separately immobilized onto two different HisTrap™ HP columns.

For the first column, 5 mL of GDH solution (0.48 mg/mL, 16.8 μM) was loaded onto a HisTrap™ HP column (1 mL) at a flow rate of 0.5 mL/min by using a peristaltic pump. Subsequently, the column was washed with 30 mL of 0.2 M phosphate buffer, pH 8.1. For the enzyme immobilization onto the second column, 5 mL of FMO3 solution (0.3 mg/mL, 5 μM) was used with the same immobilization procedure. Subsequently, the two columns were connected in series used for the following analyses.

### Enzyme immobilization—optimal pH

The connected enzyme columns were equilibrated with 8 mL of a substrate solution (1 mM NADP^+^, 1.5 mM glucose, 0.5 mM benzydamine in buffers of different pH) at a constant flow rate of 1 mL/min. 200 μL of flow-through mixed with equal volume of acetonitrile was analyzed by HPLC. The reaction was carried out at 25 °C in an incubator (Stuart orbital incubator SI50).

### Enzyme immobilization—optimal temperature

The connected enzyme columns were equilibrated with 8 mL of a substrate solution (1 mM NADP^+^, 1.5 mM glucose, 0.5 mM benzydamine in 0.2 M phosphate buffer, pH 8.1) at a constant flow rate of 1 mL/min before analyzing the flow-through by HPLC. The reaction was carried out at different temperatures ranging from 20 to 45 °C in a Stuart incubator.

### Enzyme immobilization—thermostability

The reaction was carried out at 40 °C in a Stuart incubator. The connected enzyme columns were equilibrated with 8 mL of a substrate solution (1 mM NADP^+^, 1.5 mM glucose, 0.5 mM benzydamine in 0.2 M phosphate buffer, pH 8.1) at a constant flow rate of 0.5 mL/min. Subsequently, 200 μL of flow-through solution was collected at different time point (0, 20, 40, 60, 80, 120 min), and analyzed by HPLC as described above.

### Enzyme immobilization—the effects of glycerol and histidine in substrate mixture on biotransformation

The connected enzyme columns were equilibrated with 8 mL of a substrate solution (1 mM NADP^+^, 1.5 mM glucose, 0.5 mM benzydamine, 0–12% glycerol or 0–6 mM histidine, in 0.2 M phosphate buffer, pH 8.1) at 25 °C. Subsequently, 200 μL of flow-through solution was collected and analyzed by HPLC.

### Immobilized GDH-FMO3 mediated biocatalysis generating benzydamine *N*-oxide under optimized conditions

15 mL of substrate solution (1 mM NADP^+^, 4.5 mM glucose, 1.5 mM benzydamine, in 50 mM Tris–HCl, pH 8.4) stirred by a magnetic bar was circularly flowed through (1 mL/min) the two enzyme columns by using a peristaltic pump at 35 °C. 100 μL of substrate solution was taken every 30 min, mixed with 100 μL acetonitrile, and centrifuged at 12,000×*g* for 5 min. The resulting supernatant was analyzed by HPLC.

### Whole cell catalysis—ratio optimization of GDH-BL21 and FMO3-JM109

The *E. coli* JM 109 harboring FMO3 and *E. coli* BL21 harboring GDH were used for the whole cell catalysis. The ratio of FMO3-JM109 and GDH-BL21 in reaction mixtures was optimized. The reaction mixture contained different amounts of two cell suspensions (Table 4), 1 mM NADP^+^, 10 mM glucose, 1 mM benzydamine in 0.2 M potassium phosphate (pH 8.1) buffer with a total volume of 1 mL (in a 50 mL falcon). The reaction was carried out at 35 °C, 200 rpm for 40 min, and terminated by addition of 1 mL ice-cold acetonitrile. The resulting solution was centrifuged at 12,000×*g* for 5 min and analyzed by HPLC as described above.

**Table 4:** Mixed cell suspensions of FMO3-containing *E. coli* JM109 and GDH-containing *E. coli* BL21

Ratio (W_FMO3-JM109_/W_GDH-BL21_)	20	10	6	3	1.5	1	0.6
FMO3-JM109 (g_wcw_/L)^a^	60	60	60	60	60	60	60
GDH-BL21 (g_wcw_/L)	3	6	10	20	40	60	100

a _wcw_: wet cell weight

### Whole cell catalysis—optimization of cell concentration

The optimization of cell concentration was performed by incubating different amounts of cell suspension (Table 5), 1 mM NADP^+^, 10 mM glucose, 1 mM benzydamine, in 0.2 M potassium phosphate buffer with a total volume of 1 mL. The reaction was carried out at 35 °C, 200 rpm for 40 min, terminated by 1 mL of ice-cold acetonitrile and analyzed by HPLC as described above.

**Table 5:** Different density of mixed cell suspensions

Overall cell concentration (g_wcw_/L)	20	40	80	160	320
FMO3-JM109 (g_wcw_/L)	15	30	60	120	240
GDH-BL21 (g_wcw_/L)	5	10	20	40	80

### Whole cell catalysis—the effects of citrate addition

The effects of citrate on FMO3-based whole cell catalysis was explored. The incubation mixture consisted of 30 g_wcw_/L FMO3-JM109, 10 g_wcw_/L GDH-BL21, 1 mM NADP^+^, 10 mM glucose, 1 mM benzydamine, different concentration of citrate (0–100 mM), in phosphate buffer (pH 8.1) with a total volume of 1 mL. The reaction was carried out at 35 °C, 200 rpm for 40 min, and analyzed by HPLC as described above.

### Whole cell catalysis—optimization of substrate concentration

The reaction mixture contained 30 g_wcw_/L FMO3-JM109, 10 g_wcw_/L GDH-BL21, 1 mM NADP^+^, 10 mM glucose, different concentration of benzydamine ranging from 1 to 10 mM, in phosphate buffer (pH 8.1) with a total volume of 1 mL. The reaction was carried out at 35 °C, 200 rpm for 4 h, and analyzed by HPLC as described above.

### Whole cell catalysis—optimization of NADP^+^ concentration

The reaction mixture contained 30 g_wcw_/L FMO3-JM109, 10 g_wcw_/L GDH-BL21, 10 mM glucose, 4 mM benzydamine, different concentration of NADP^+^ (1–20 mM), in phosphate buffer (pH 8.1) with a total volume of 1 mL. The reaction was carried out at 35 °C, 200 rpm for 5 h, and analyzed by HPLC as described above.

### FMO3-based whole cell biotransformation generating benzydamine *N*-oxide under optimized conditions

The reaction mixture contained 30 g_wcw_/L FMO3-JM109, 10 g_wcw_/L GDH-BL21, 10 mM Glucose, 4 mM benzydamine, 5 mM NADP^+^, in phosphate buffer (pH 8.1) with a total volume of 10 mL (in a 50 mL shake flask). The reaction was carried out at 35 °C, 200 rpm. At different time point, 100 μL of sample was taken and mixed with 100 μL of ice-cold acetonitrile, centrifuged at 12,000×*g* for 5 min. The resulting supernatant was analyzed by HPLC as described above.

### Bioconversion of tamoxifen to its *N*-oxide metabolites by the two easy-to-perform approaches

For the *N*-oxidation of tamoxifen. The final concentration of tamoxifen (Stock solution: 100 mM tamoxifen in ethanol) used for whole cell system and immobilization system was 1 mM and 0.5 mM respectively while keeping all other conditions constant. Samples were taken at different time-points, mixed with equal volume of ice-cold acetonitrile and analyzed by HPLC. A mobile phase of 40% acetonitrile and 60% formic acid (0.1%) in water was used to separate the tamoxifen and its *N*-oxide, and the effluent was monitored at 276 nm.

## Additional file

**Additional file 1: Fig. S1.** UV–vis absorption spectra of the purified (a) FMO3 and (b) GDH. (c) 12.5% SDS-PAGE analysis of FMO3 and GDH. Lane 1: Molecular weight marker, Lane 2: FMO3-containing whole cell proteins, Lane 3: The purified FMO3 (5 μg of total protein), Lane 4: GDH-containing whole cell proteins, Lane 5: The purified GDH (3 μg). **Fig. S2.** The His_6_-tagged GDH and FMO3 enzymes were separately loaded onto two different HisTrap™ HP columns, followed by connection in series.

## Data Availability

All data generated or analyzed during this study are included in this published article and its additional files.

## Abbreviations

GDH: glucose dehydrogenase
FMO3: flavin-containing monooxygenase isoform 3
FMO2: flavin-containing monooxygenase isoform 2
IPTG: isopropyl β-d-thiogalactoside
NADP^+^: nicotinamide adenine dinucleotide phosphate, oxidized form
NADPH: nicotinamide adenine dinucleotide phosphate, reduced form
HPLC: high performance liquid chromatography
WCW: wet cell weight
