# Expression of Concern to: Drug metabolite synthesis by immobilized human FMO3 and whole cell catalysts

**DOI:** 10.1186/s12934-020-01327-y

**Published:** 2020-03-25

**Authors:** Chongliang Gao, Tingjie Zheng

**Affiliations:** 1 Department of Life Sciences and Systems Biology, University of Torino, Via Accademia Albertina 13, 10123 Turin, Italy; 2 Department of Cultures, Politics and Society, University of Torino, Lungo Dora Siena 100, 10153 Turin, Italy

## Expression of Concern: Microb Cell Fact (2019) 18:133 10.1186/s12934-019-1189-7

The Editor-in-Chief is issuing an Editorial Expression of Concern as after publication of this article [1], it has come to light that the biological materials were used and the experiments were conducted without proper authorisation from the laboratory where these data were obtained. In addition, at the time when the research was performed, the first author was a PhD student in the Department of Life Sciences and Systems Biology, University of Torino, and the second author was a master’s student in the Department of Culture, Politics and Society, University of Torino. The affiliation of the second author has therefore been corrected. However, there is no evidence suggesting that the research presented in the article is scientifically unsound.

Neither of the authors agree to this Expression of Concern.
