# Supplementary material for: Drug metabolite synthesis by immobilized human FMO3 and whole cell catalysts
Source: Microb Cell Fact. 2019 Aug 12;18:133. doi: 10.1186/s12934-019-1189-7 (PMC6691536; doi:10.1186/s12934-019-1189-7)
Supplement: Supplementary file 1 — Additional file 1: Fig. S1. UV–vis absorption spectra of the purified (a) FMO3 and (b) GDH. (c) 12.5% SDS-PAGE analysis of FMO3 and GDH. Lane 1: Molecular weight marker, Lane 2: FMO3-containing whole cell proteins, Lane 3: The purified FMO3 (5 μg of total protein), Lane 4: GDH-containing whole cell proteins, Lane 5: The purified GDH (3 μg). Fig. S2. The His6-tagged GDH and FMO3 enzymes were separately loaded onto two different HisTrap™ HP columns, followed by connection in series. [file 12934_2019_1189_MOESM1_ESM.docx]

Fig. S1


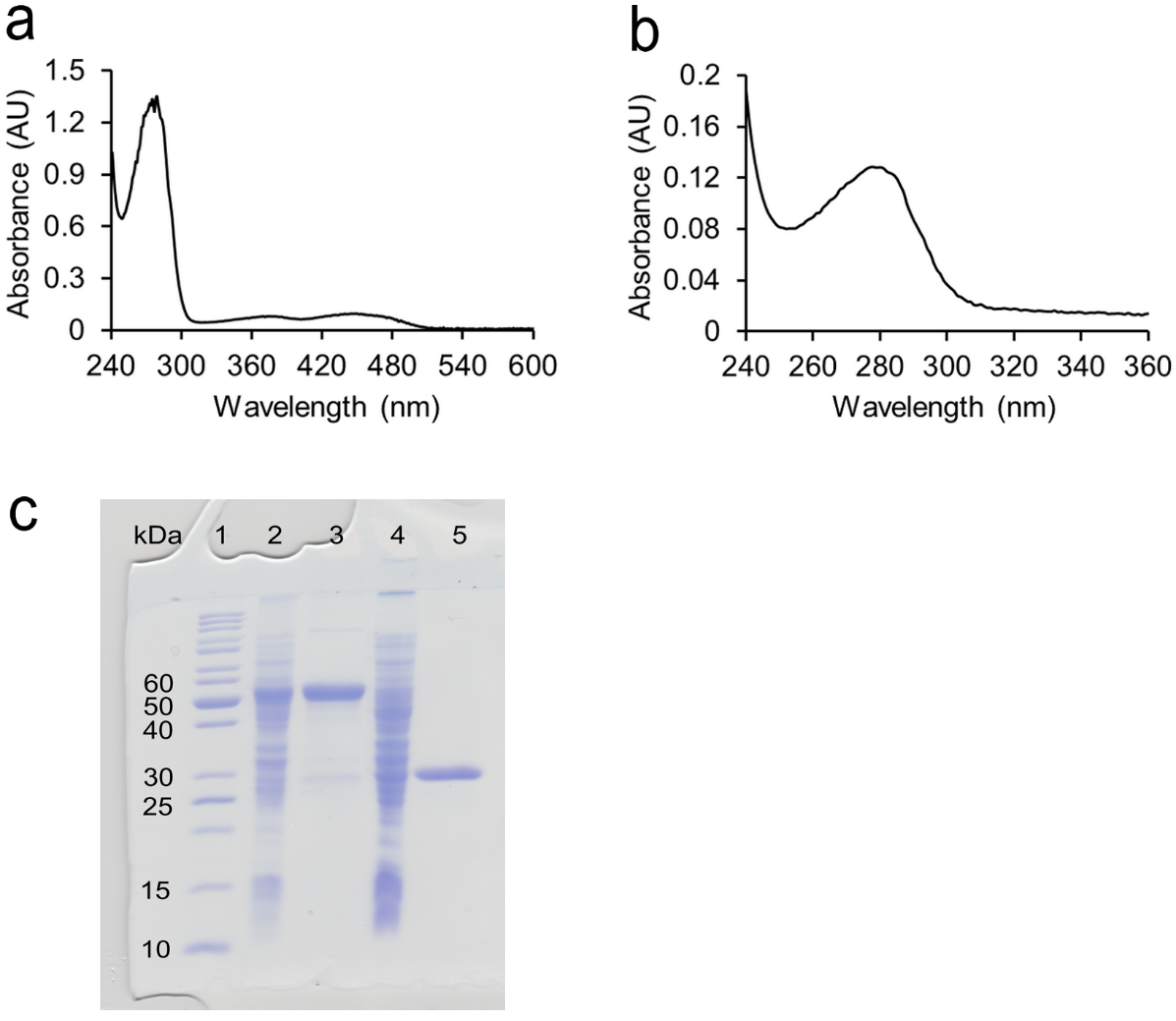


Fig. S1. UV-vis absorption spectra of the purified (a) FMO3 and (b) GDH. (c) 12.5% SDS-PAGE analysis of FMO3 and GDH. Lane 1: Molecular weight marker, Lane 2: FMO3-containing whole cell proteins, Lane 3: The purified FMO3 (5 μg of total protein), Lane 4: GDH-containing whole cell proteins, Lane 5: The purified GDH (3 μg).

Fig. S2


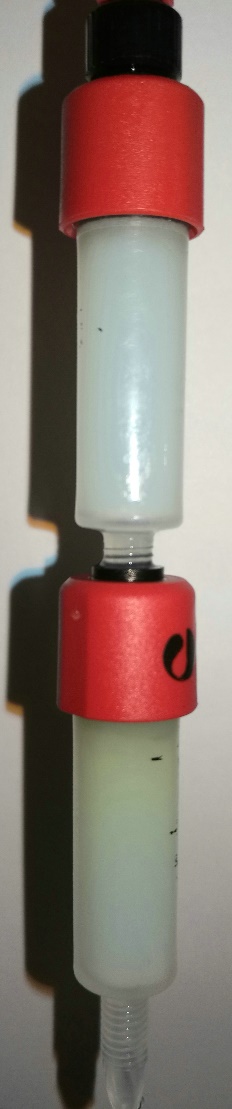


The FMO3-binding column

The GDH-binding column

Fig. S2. The His_6_-tagged GDH and FMO3 enzymes were separately loaded onto two different HisTrap^TM^ HP columns, followed by connection in series.
